# Management and monitoring strategies for severe cerebral malaria: a guide for the intensivist

**DOI:** 10.1186/s13613-025-01584-3

**Published:** 2025-10-08

**Authors:** Sonila Vathi, Alberto Corriero, Edith Elianna Rodríguez, Anthony Moreau, Elisa Gouvea Bogossian, Marta Baggiani, Maya Hites, Romain Sonneville, Fabio Silvio Taccone, Michele Salvagno

**Affiliations:** 1 Department of Intensive Care, Hôpital Universitaire de Bruxelles (HUB), Université Libre de Bruxelles, Brussels, Belgium; 2 Anesthesia and Intensive Care Department, University of Turin, Turin, Italy; 3 Department of Interdisciplinary Medicine-Intensive Care Unit Section, University of Bari Aldo Moro, Bari, Italy; 4 School of Medicine and Health Sciences, Universidad del Rosario, Bogotá, Colombia; 5 Neurointensive Care Unit, Neuroscience Department, Fondazione IRCCS San Gerardo dei Tintori, Monza, Italy; 6 Clinic of Infectious Diseases, HUB, Université Libre de Bruxelles, Brussels, Belgium; 7 INSERM UMR1137, IAME, Department Intensive Care Medicine, Hôpital Bichat Claude Bernard, AP-HP, Université Paris Cité, Paris, France

**Keywords:** Malaria, Cerebral, Seizures, Intracranial hypertension, Electroencephalography, Chemotherapy, Adjuvant, Artesunate, Blood-brain barrier, Critical care, Anticonvulsants, Biomarkers

## Abstract

Severe malaria, caused by *Plasmodium falciparum*, poses a critical public health challenge, with cerebral malaria (CM) representing its most severe and life-threatening neurological manifestation. Defined by impaired consciousness (Glasgow Coma Score < 11) after the exclusion of other causes of encephalopathy, CM remains a critical condition with a mortality rate of 15–25% and long-term neurological sequelae in survivors. CM pathogenesis involves parasitized erythrocyte sequestration in cerebral microvasculature, immune hyperactivation, blood-brain barrier disruption, and cerebral edema, potentially leading to elevated intracranial pressure (ICP) and cerebral ischemia. These processes culminate in severe neurological injury, emphasizing the importance of ICP management in minimizing secondary brain damage. Neuromonitoring (NM) strategies, including invasive and non-invasive techniques, are critical yet underutilized in adults with CM due to limited evidence and logistical challenges. Treatment relies on antimalarial therapy, with intravenous artesunate as the first-line drug, supported by targeted interventions to manage seizures and systemic complications. Adjunctive therapies remain experimental, with no proven benefit in routine care. Emerging evidence from pediatric studies offers valuable insights, though significant gaps in adult-focused research persist. This review, which examines severe CM pathophysiology, clinical manifestations, and management, focusing on adult populations, underscores the need for tailored NM approaches, protocolized management strategies, and further investigation to improve outcomes in adults with CM, advocating for a multidisciplinary approach within the intensive care setting.

## Introduction

Malaria is an infectious disease caused by Plasmodium parasites, transmitted to humans by female *Anopheles* mosquitoes. Five species can infect humans (*P. falciparum*, *P. malariae*, *P. ovale*, *P. vivax*, and *P. knowlesi*) [1]. Every year, malaria is responsible for the death of at least half a million people worldwide [2], ranking among the top public health concerns according to the World Health Organization (WHO). Factors such as acidemia, hemolytic anemia and the sequestration of infected erythrocytes (IE) in the microvasculature of multiple organs contribute to the development of severe forms of malaria, which occurs when the disease involves the cerebral, cardiovascular, renal, or placental circulation [3]. Individuals at the most significant risk of succumbing to malaria are those living in the WHO African region, and most particularly those younger than 5 years of age, or individuals visiting friends and family in the WHO African region who do not take adequate chemoprophylaxis [2].

Cerebral malaria (CM) is the most severe neurological complication of *Plasmodium falciparum* malaria. It is clinically defined by a severe alteration of mental status (“unarousable coma”) in the context of definite malarial infection after the exclusion of other causes (including post-ictal states, hypoglycemic coma, and meningitis) [4]. Typically, this condition needs management in the intensive care unit (ICU) [5–7]. In malaria-endemic areas, around 575,000 cases are diagnosed annually [8]; while the vast majority of reported cases involve children, particularly in sub-Saharan Africa, adult cases, especially among travelers or individuals in low-transmission regions, represent a growing concern. Despite improvement in patients’ management, the prognosis of CM remains poor, with an estimated mortality rate of around 20% and poor neurological outcome exceeding 25% of the cases [9]. Acute management should focus on detecting systemic complications, seizures and/or intracranial hypertension (IH). Invasive intracranial pressure (ICP) monitoring may be unavailable or contraindicated due to thrombocytopenia, making non-invasive options like cerebral ultrasound preferable [10].

This review aims to assess the existing evidence and to provide some guidance for adult CM management in the ICU setting.

## Methods

This article is a narrative review, aiming to synthesize current knowledge on CM, with a particular focus on the intensive care setting, in particular with the use of neuromonitoring. As such, the review did not follow the PRISMA guidelines and was not intended as a systematic review. A structured literature search was conducted across PubMed, Scopus and Web of Science databases for articles published in English between January 2000 and February 2025. The following keywords were used alone or in combination: “cerebral malaria,” “Plasmodium falciparum,” “intracranial hypertension,” “neurocritical care,” “EEG malaria,” “TCD malaria,” “optic nerve sheath diameter,” and “biomarkers malaria.” We prioritized clinical studies, expert opinions, consensus statements, and WHO guidelines. Reference lists of key papers were screened to identify additional sources.

The neurological manifestations of *P. falciparum* malaria vary significantly between adults and children [11, 12]. However, there is a notable imbalance in the literature, with a predominance of studies focusing on children with CM. Accordingly, emphasis was placed on transparency and relevance, particularly regarding findings related to adult CM and the applicability of neuro-monitoring strategies across diverse clinical settings.

## Pathophysiology

The limited availability of in vitro cell culture models that accurately replicate the molecular mechanisms underlying CM in humans has significantly hindered progress in understanding the disease pathophysiology. The main hypotheses underlying brain damage secondary to *P. falciparum* infection rely on interconnected pathological events. The process begins with the sequestration of infected erythrocytes (IEs) within the cerebral microvasculature, resulting in capillary perfusion and causing secondary ischemia [13]. IEs express surface proteins, such as *P. falciparum* erythrocyte membrane protein 1 (PfEMP1), which bind to endothelial receptors like ICAM-1, VCAM-1, and CD-36 [14]. This binding activates endothelial cells, triggering the coagulation cascade and microthrombus formation, worsening ischemia [15]. IEs may also accumulate in draining veins and sinuses, especially at the rostral confluence, contributing to brain edema [16]. Thrombocytopenia and disseminated intravascular coagulation (DIC) further increase the risk of cerebral hemorrhage [17]. PfEMP1 interaction with host molecules such as EPCR and thrombospondin exacerbates endothelial dysfunction and microcirculatory impairment [18]. The potential role of extracellular vesicles carrying parasitic components in enhancing cerebral involvement remains poorly studied [19].

These phenomena are accompanied by a dysregulated inflammatory response marked by excessive release of pro-inflammatory cytokines including TNF-α, IL-1β, and IFN-γ [20]. While critical for controlling parasitemia, these cytokines induce endothelial activation, increase blood-brain barrier (BBB) permeability, and contribute to vascular damage [21]. Cerebral edema develops via capillary obstruction from IEs and cytokine-mediated BBB dysfunction, causing both cytotoxic and vasogenic edema [14]. Elevated cytokines promote immune cell recruitment, leukocyte adhesion, and oxidative stress, resulting in neuronal apoptosis and tissue injury [22]. Neutrophil activation leads to the formation of extracellular traps (NETs) that, while aiding parasite control, also promote inflammation and tissue damage [23]. Upregulated endothelial receptors may further enhance IE sequestration [23]. Platelets bind to IEs and endothelium via CD36 and ICAM-1, contributing to microthrombi and impaired perfusion [18]. The resulting edema raises intracranial pressure (ICP), worsening perfusion and leading to seizures, coma, or fatal brain herniation [24, 25].

Neurodegenerative pathways, observed particularly in hemozoin-exposed models, may underlie prolonged neurological sequelae in CM survivors [24]. Notably, neurocognitive symptoms have also been reported after non-severe malaria and even in asymptomatic carriers [25, 26]. Microglia contribute to the neuroinflammatory cascade by releasing TNF-α, IL-1β, nitric oxide, and ROS, further disrupting the BBB and promoting neurotoxicity [27]. Another relevant aspect is the functional exhaustion of innate immune cells, especially granulocytes. Phagocytosis of hemozoin-rich vacuoles depletes cellular reserves and antimicrobial capacity, leading to immune suppression [28]. This may explain the high rate of secondary bacterial infections in children with CM and in adults with severe imported malaria, where co-infections reach up to 30% [10].

**Fig. 1:**
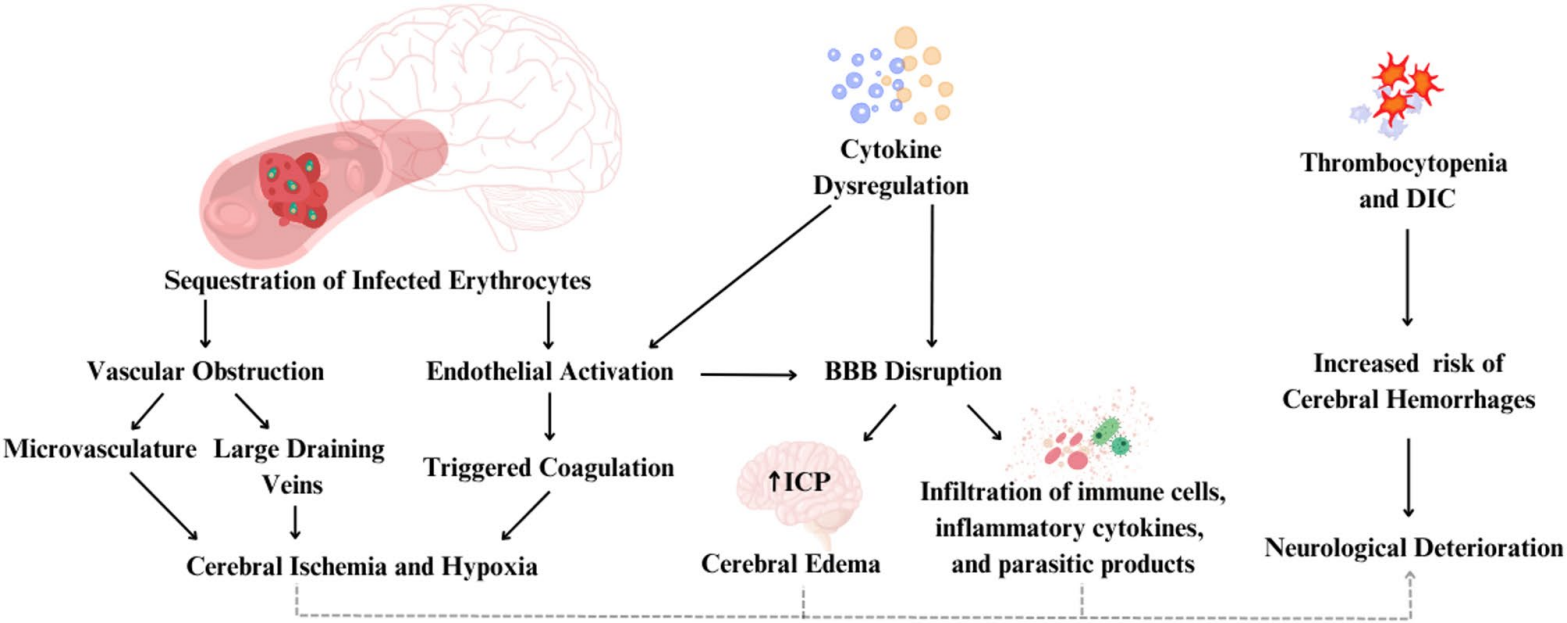
Main mechanisms involved in the pathogenesis of cerebral malaria. (1) Flow disturbances caused by sequestration of infected erythrocytes (IE) in capillaries; (2) Excessive cytokine production; (3) Brain blood barrier (BBB) damage; (4) Cerebral Edema and increased intracranial pressure (ICP). *DIC* disseminated intravascular coagulation

Figure 1 summarizes CM pathophysiology based on IE sequestration and systemic inflammation. However, these mechanisms do not fully explain the relatively low rate of neurological deficits in survivors, nor the occurrence of cognitive symptoms in mild or asymptomatic malaria. A complementary “metabolic” hypothesis has been proposed, suggesting that liver dysfunction and BBB disruption allow neurotoxins (e.g., ammonia) to reach the brain and trigger CM symptoms [29]. Host genetics also influences CM susceptibility. The sickle cell trait (HbAS) is protective against severe malaria, likely by altering IE adherence [30]. Likewise, Southeast Asian ovalocytosis reduces cerebral sequestration of IEs and may protect against CM [31]. These genetic traits modulate host-pathogen interactions, influencing disease severity.

## Clinical presentation

CM may present either as a neurological complication in a patient already diagnosed or suspected of having malaria, or as an acute coma or seizure in individuals without prior malaria diagnosis, particularly in non-endemic settings. In all patients presenting with fever or altered mental status who have lived in or recently returned from endemic areas, malaria should be considered in the differential diagnosis [10]. The clinical spectrum is broad, with cyclic fever and rigors in more than 50% of cases. Other infectious diseases (e.g., Zika, Chikungunya, dengue, leptospirosis, typhoid, yellow fever) must also be ruled out. Diagnosis relies on identifying parasites on blood smear microscopy (the gold standard), which should be repeated within 48 h if initial results are negative but suspicion remains high.

Severe malaria encompasses the involvement of multiple systems. The WHO and the Centers for Disease Control and Prevention (CDC) define it as the presence or detection of any species of *Plasmodium* along with evidence of organ failure (Table 1) [32]. Cerebral malaria is diagnosed when patients exhibit an inability to localize a painful stimulus (e.g., in general, a Glasgow Coma Scale, GCS < 11 or Blantyre Scale >3 in children), along with peripheral asexual *P. falciparum* parasitemia, and no other identifiable causes of encephalopathy (including post-ictal states, hypoglycemic coma, and meningitis). While no specific threshold of parasitemia is required for diagnosis, *P. falciparum* hyper-parasitemia, defined as parasitemia >10%, is considered a marker of severe disease and increases the likelihood of complications, including cerebral involvement [33]. In non-endemic settings, a parasitemia threshold of ≥ 4% is often used as a cut-off for severe malaria, as recognized in the 2024 WHO guidelines [34]. The asexual forms of the parasite on a peripheral blood smear are characteristic features of the disease in unrousable coma patients after the exclusion of common confounders [4].

Seizures are a frequent and critical feature of CM, occurring in up to 80% of pediatric cases [35] and approximately 15–20% of adults [36]. These seizures may manifest as generalized tonic-clonic seizures, focal motor seizures, or even subtle or subclinical seizure activity detectable only through electroencephalography (EEG). Untreated seizure activity can exacerbate neuronal injury and worsen IH, necessitating prompt recognition and treatment [37]. Moreover, post-malarial epilepsy has been observed in pediatric survivors of CM, suggesting a long-term risk of neurological sequelae following severe disease [38].

Focal neurological deficits, although less common, may occur in CM and are usually indicative of localized ischemic or hemorrhagic events secondary to vascular occlusion or microthrombi formation [39]. These deficits may include unilateral weakness, facial droop, or localized sensory loss, which require careful clinical assessment to differentiate from postictal states or other neurological conditions. Some patients, particularly children, may experience persistent neurological sequelae following CM, collectively referred to as post-malarial neurological syndrome (PMNS). PMNS can manifest as cognitive impairments, attention deficits, motor dysfunction (such as tremors and ataxia), behavioral disturbances, and, in some cases, epilepsy. While many deficits resolve over time, others may persist for years, requiring long-term monitoring and rehabilitation [40].

Signs of brainstem involvement, such as abnormal pupillary reflexes, respiratory irregularities, or impaired cranial nerve function, are not well-documented in clinical cases of cerebral malaria. While experimental studies, such as those highlighting the role of CD8^+^ T cells in disrupting the BBB and contributing to localized inflammation [41], suggest the potential for brainstem impairment in such patients, there is currently no direct evidence supporting this hypothesis in human cases. This highlights a significant gap in the literature, and further research is needed to explore whether and how brainstem involvement contributes to the pathophysiology and clinical presentation of CM.

Together with various degrees of disorders of consciousness, patients with CM can also exhibit signs of meningitis or cerebellar ataxia [37]. Delayed cerebellar ataxia has been specifically reported in post-malarial cases, occurring days to weeks after recovery and typically resolving spontaneously [42]. Moreover, visual disturbances can also occur in patients with CM. While blurred vision can be a clinical manifestation of intracranial hypertension, retinopathy is a distinct complication associated with CM. This retinopathy, particularly prevalent in children with a high parasite burden, is characterized by retinal whitening, vessel color changes, and/or hemorrhages [43].

**Table 1:** Clinical presentation of severe malaria in adults

Compromised system	Clinical manifestations
Cardiovascular	Systolic arterial blood pressure < 90 mmHg and/or presence of peripheral signs of circulatory failure; shock; need for vasopressors and lactate levels > 2 mmol/L
Respiratory	Requirement of mechanical ventilation or non-invasive ventilation, respiratory failure (PaO_2_/FiO_2_ < 300 mmHg and/or respiratory rate > 30/min), hypoxemia (PaO_2_ < 60 mmHg and/or SpO_2_ < 92% of ambient air) and interstitial and/or alveolar infiltrates in images, pulmonary edema.
Renal	Oliguria and/or blood creatinine level > 265 mmol/L or urea > 20 mmol/L
Metabolic	Hypoglycemia (< 2.2 mmol/L), acidosis (plasma bicarbonate levels < 15 mmol/L or pH < 7.35) and hyperlactatemia (> 2 mmol/L)
Coagulation	Recurrent or prolonged bleeding from nose, gums, venipuncture sites, melena, hematemesis
Hepatic	Jaundice or total bilirubin > 50 mmol/l
Neurological	Obnubilation, confusion, drowsiness, prostration, disorders of consciousness, seizures
Hematological	Anemia (hemoglobin < 7 g/dL Hct < 20%))
Endocrine/electrolyte	Hyponatremia (< 130 mmol/L), hypernatremia (> 150 mmol/L), hypokalemia (< 3.5 mmol/L), and other electrolyte imbalances. May be associated with SIADH or adrenal insufficiency. These alterations can exacerbate neurological symptoms and require correction.

(Adapted from F. Bruneel et al./Médecine et Maladies Infectieuses 50 (2020) 213–225)

*SBP* Systolic Blood Pressure,* RF* Respiratory Failure,* PaO₂* Partial Pressure of Oxygen,* FiO₂* Fraction of Inspired Oxygen,* SpO₂* Peripheral Oxygen Saturation,* Cr* Creatinine,* HCO*_3_^*−*^ Bicarbonate,* Hb* Hemoglobin,* Hct* Hematocrit,* SIADH* Syndrome of inappropriate antidiuretic hormone secretion

## How to monitor the brain of a patient with severe cerebral malaria?

### Detection of cerebral secondary damage of systemic origin

In CM, systemic insults often contribute to secondary brain injury. The GHOST-CAP acronym provides a structured approach for bedside monitoring and intervention [44]. It includes glycemia (G), as both hypo- and hyperglycemia may worsen neuronal injury and require prompt correction; hemoglobin (H), since severe anemia impairs oxygen delivery and may require transfusion; oxygenation (O), to detect and treat hypoxemia that can lead to cerebral hypoxia; sodium (S), as hyponatremia is frequent and associated with cerebral edema and seizures; temperature (T), since fever raises metabolic demands and should be managed aggressively; comfort (C), to minimize agitation and pain, which can raise ICP; arterial pressure (A), to ensure sufficient cerebral perfusion pressure (CPP); and PaCO₂ (P), as hypercapnia and hypocapnia can alter cerebral blood flow. Each parameter represents a modifiable factor that must be actively monitored and managed to prevent secondary neurological deterioration.

### Brain imaging

Brain CT-scan and magnetic resonance imaging (MRI) are widely used to assess brain edema [45]. Brain CT-scan should complete the initial work-up of all patients with severe CM to identify the presence of ischemic or hemorrhagic lesions, in particular in unconscious patients or those with focal deficit [46]. In some patients, CT may reveal minor ischemic lesions and minor edema (intermediate risk), while in severe cases cerebral edema or large territorial ischemic/hemorrhagic lesions may be evident (high risk) [47]. Data on CT perfusion and CT angiography are scarce in this setting; however, these technologies might be, as in other acute cerebrovascular disease, valuable tools. In a study involving 21 adults, anomalies detected on CT scans were found to be correlated with both the GCS on admission and mortality rates [48]. Cerebral edema was observed in eight patients, and two of them died.

**Fig. 2:**
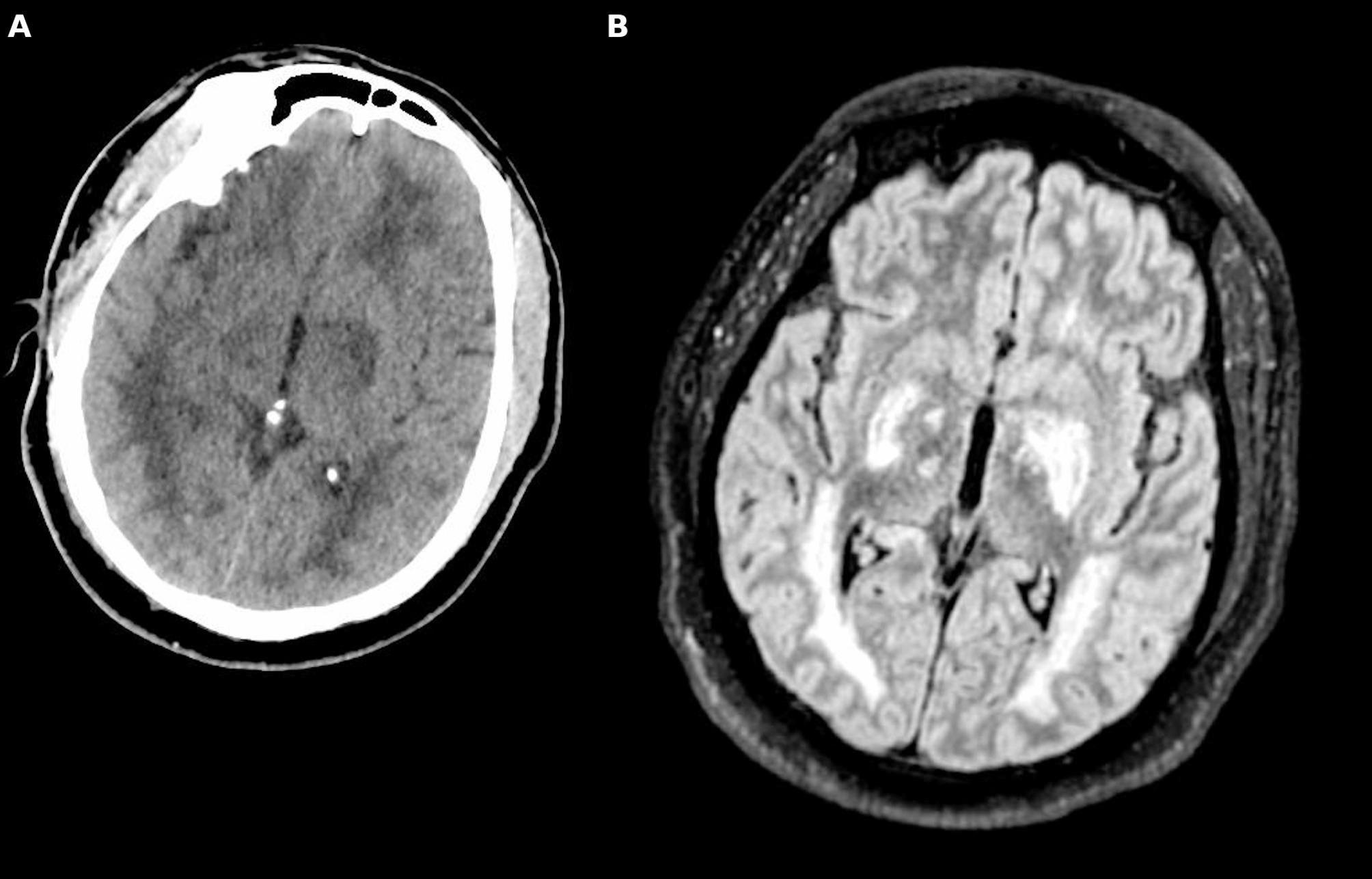
Brain imaging in cerebral malaria. Cerebral CT-scan (**A**) and Magnetic Resonance Imaging (MRI) Fluid Attenuated Inversion Recovery (FLAIR - **B**) sequences showing diffuse involvement of the white matter, thalamus and globus pallidus, in a patient with cerebral malaria

MRI provides greater detail and has shown different patterns in adults and children: cytotoxic edema, bilateral thalamic hyperintensities, corpus callosum, and occipital white matter lesions are commonly reported in adults [49, 50] (Fig. 2). In contrast, children often show signs of vasogenic edema and increased brain volume [51, 52]. Notably, in some adult series from Thailand [53], brain swelling was due to hyperemia rather than typical edema.

Additionally, posterior reversible encephalopathy syndrome (PRES) may be identified by MRI in cases of CM [54]. Although systematic data on its prevalence are lacking, PRES has been described in several adult case series and may reflect blood–brain barrier dysfunction and impaired cerebral autoregulation secondary to systemic inflammation [54]. A stepwise diagnostic approach, beginning with CT-scan imaging as the initial modality and followed by MRI in selected cases, such as those presenting with brainstem or cerebellar signs, is advisable when feasible.

### Identifying patients at risk of elevated intracranial pressure

In central nervous system infections, screening for IH is currently underused [55, 56]; this may include the recognition of clinical signs (e.g., persistent coma, bradycardia, hypertension, irregular respiration) or indirect signs from neuroimaging (e.g., cerebral edema, midline shift). Notably, IH has been identified as a significant predictor of morbidity and mortality in CM, with an 84% prevalence in fatal cases compared to 27% in non-fatal cases [8, 57]. Elevated ICP contributes to brain damage by inducing global ischemia, which reduces cerebral perfusion pressure (CPP) or compromises the basal cerebral arteries during trans-tentorial herniation [58].

Most of the available literature on IH comes from studies conducted on African infants, who are now recognized as one of the most vulnerable populations in this endemic zone [58]. However, no guidelines and only limited data are available for the use of invasive ICP monitoring, nor of additional brain tissue oxygen pressure (PbtO_2_) monitoring, in this context, as it has been proposed for head trauma [58–60]. Moreover, considering the coagulation disorder linked to severe malaria [61], the routine use of invasive ICP monitoring in CM patients is often contraindicated. In children, lumbar puncture (LP) has been shown to be feasible to measure ICP, even in comatose patients, without additional risks [62]. However, LP in adults may be more controversial, as there is no data available on its safety, and it might be contraindicated, especially if signs of IH are detected on imaging.

Non-invasive monitoring of ICP represents a crucial advancement in the management of CM, particularly in resource-limited settings where invasive techniques are often unavailable. Trans-cranial Doppler (TCD) is a widely accessible non-invasive method for assessing cerebral hemodynamics, but its application in CM is limited by the need for trained operators, especially in low- and middle-income countries (LMICs). In practice, TCD can be utilized to measure systolic (sBFV), diastolic (dBFV), and mean blood flow velocity (mBFV) in the middle cerebral artery, providing critical data on cerebral perfusion and potential vascular disturbances. This technique can also derive indices such as the pulsatility index (PI) and resistance index (RI), which are instrumental in potentially identifying hyperemia, microvascular obstruction, low-flow states, or vasospasm, e.g., pathophysiological states commonly observed in CM [63–65]. For effective clinical application, TCD can be scheduled at regular intervals to monitor changes in cerebral hemodynamics over the course of treatment, thereby helping to track disease progression or improvement. Additionally, TCD findings, such as elevated PI (>1.4) or reduced flow velocities, which correlate with increased ICP, can inform decisions regarding therapeutic interventions to reduce ICP and/or increase CPP [65]. Markedly reduced mBFV with dBFV < 20 cm/s have been associated with severe IH and poor cerebral perfusion [63]. In a study, 160 children with CM were examined, revealing four distinct flow patterns: hyperemia (26%), microvascular obstruction (22%), low flow (28%) and vasospasm (13%) [66]. This heterogeneity suggests that different mechanisms may coexist and CM may not always lead to ICP elevation through the same pathway of flow disturbance. Additionally, sonographic abnormalities identified through TCD have been linked to lateralizing deficits and severe IH, demonstrating a significant linear correlation between CPP and cerebral blood flow velocities [65].

Optic nerve sheath diameter (ONSD) is a relatively low-cost and portable technique, making it suitable for resource-limited settings, although it also requires adequate training, and interpretation may vary significantly between operators, limiting reproducibility. This method relies on the anatomical principle that elevated ICP leads to distension of the optic nerve sheath, which can be measured 3 mm posterior to the globe using high-frequency ultrasound probes. In pediatric studies, ONSD measurements exceeding specific thresholds (e.g., >5.0 mm in younger children, gradually increasing with age) have demonstrated good sensitivity and specificity in predicting elevated ICP (i.e. >20 mmHg) [67–69]. In adults, a threshold of >5.8 mm has been widely used to predict IH after head trauma [70]. These variations reflect anatomical differences between pediatric and adult populations, emphasizing the importance of age-specific thresholds for accurate assessment. However, at least two studies [57, 71] highlighted conflicting findings regarding the correlation between ONSD and ICP in CM. In particular, a study conducted in 112 children with CM found no significant correlation between ONSD and cerebrospinal fluid (CSF) opening pressure during LP [68]. Conversely, one study on 87 children with CM reported that increased ONSD measurements were associated with worse neurological outcomes and higher risk of death [69]. These findings underscore the need for larger studies to establish the predictive value of ONSD in CM patients [55].

Quantitative automated pupillometry provides a non-invasive and objective method for assessing neurophysiological responses and detecting early signs of neurological deterioration. This technique measures pupillary variables, including the Neurological Pupil Index (NPi), a standardized index derived several metrics of pupillary function during a light stimulation [72]. Abnormal findings in automated pupillometry have been associated with elevated ICP in various critical care contexts and may serve as an adjunct tool for monitoring IH in CM. For example, a study in traumatic brain injury has shown that abnormal NPi scores (< 3) and reduced constriction velocities correlate with increased ICP and poor neurological outcomes [73]. One case series demonstrated that early detection of pupillary abnormalities facilitated timely interventions, such as osmotherapy or surgical decompression, potentially mitigating neurological damage [74]. These findings suggest that similar applications could be extended to CM; however, no data on its use in CM are currently available.

Retinal assessment is a key diagnostic tool for evaluating cerebral microcirculation in patients with CM, particularly in endemic regions where its utility has been recognized since 2006 [10]. Specific retinal abnormalities, such as whitening, changes in vessel color, and hemorrhages, are highly indicative of malaria and play a crucial role in distinguishing malarial from non-malarial coma. Notably, while often present, papilledema is not specific to malaria and may indicate other coma-inducing conditions [75]. Retinal assessment through fundoscopy remains an effective and accessible tool for detecting direct signs of IH and distinguishing coma from other causes [76]. Its integration into diagnostic protocols, both in well-equipped and resource-limited settings, could minimize misdiagnosis and improve patient outcomes. However, accurate interpretation of malarial retinopathy requires dedicated training and experience, particularly for distinguishing specific features such as retinal whitening, vessel discoloration, and hemorrhages from other causes of coma-associated fundus changes.

**Fig. 3:**
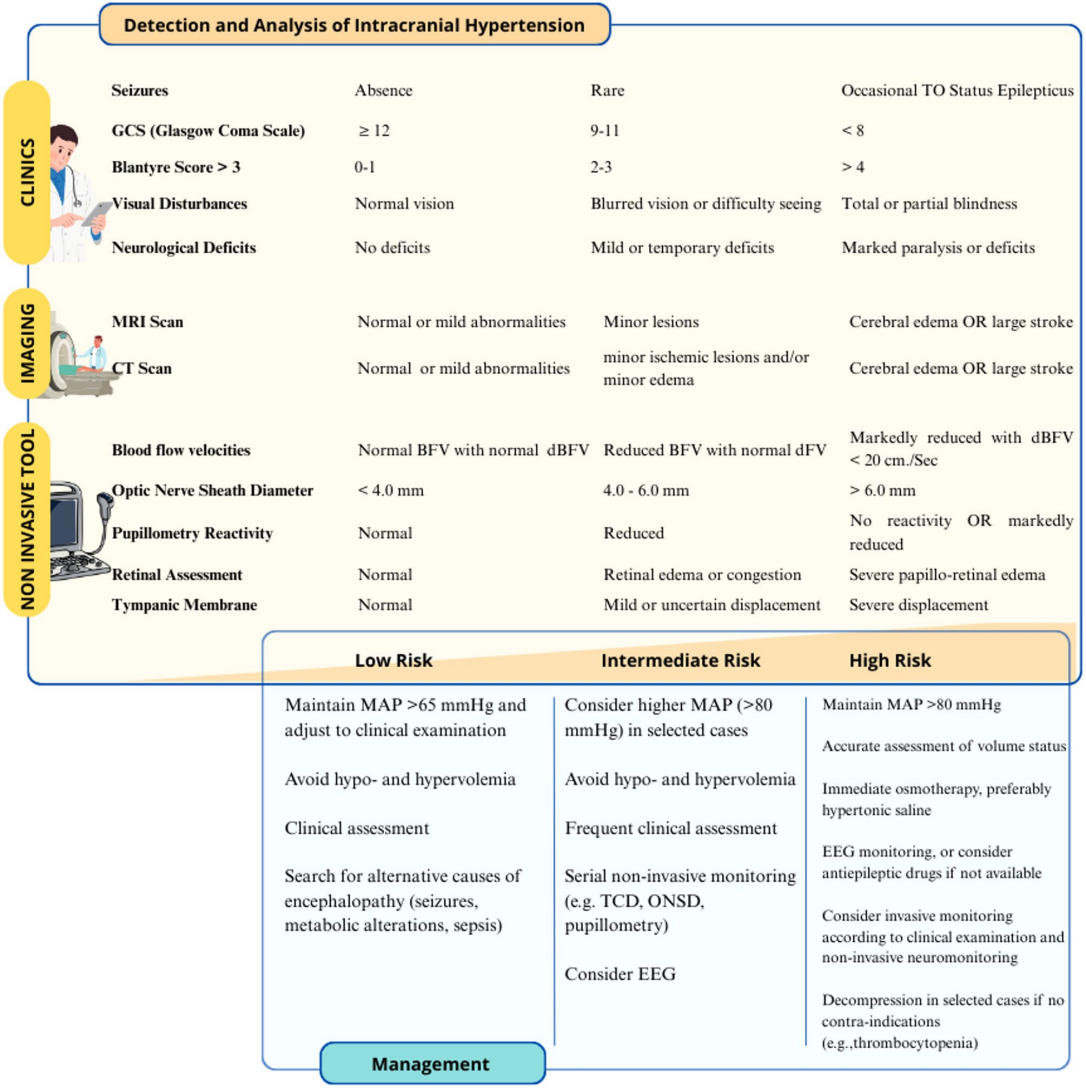
Multimodal risk stratification for intracranial hypertension in cerebral malaria. *GCS* Glasgow Coma Scale, *BFV* blood flood velocity, *dBFV* diastolic blood flow velocity. Focal deficits may not be present in the context of increased intracranial pressure due to global edema and diffuse injury

An innovative, non-invasive, bedside applicable method for ICP monitoring is tympanic membrane displacement (TMD), but it is currently limited to pediatric studies [77]. The technique involves identifying minute movements of the tympanic membrane induced by pulsatile pressure variations transmitted through the auditory canal, which correspond to variations in intracranial pressure [77]. A risk-stratified approach to detect and manage IH in CM patients is presented in Fig. 3.

### Seizures detection

EEG may contribute to the management of CM by detecting both clinical and subclinical seizures, localizing epileptiform discharges, and identifying patterns with prognostic significance [78, 79], although most supporting data derive from non-malarial coma. Continuous EEG can enhance the detection of non-convulsive seizures and guide targeted anticonvulsant therapy [80–82]; however, its implementation may be constrained by the requirement for continuous availability of specialized neurophysiological expertise.

### Biomarkers

Several biomarkers have emerged as tools in diagnosing and managing CM, offering insights into its pathophysiology and severity. Among them, endothelial activation markers such as angiopoietin-2 (Ang-2) [83], soluble intercellular adhesion molecule-1 (sICAM-1) [84], and von Willebrand factor (VWF) [85] reflect endothelial dysfunction and BBB disruption, which are central to CM pathogenesis. Elevated Ang-2 levels and decreased angiopoietin-1 (Ang-1) have been associated with vascular instability and disease severity [86]. These markers, although not yet widely available, are among the most promising in terms of potential clinical application.

Other biomarkers remain largely experimental and require further validation. Circulating microparticles (MPs), derived from platelets, monocytes, and endothelial cells, have been implicated in coagulation and inflammation, serving as indirect indicators of endothelial injury and neurological dysfunction in CM [87]. However, MP elevation is not specific and can occur in other thrombocytopenic or neurologically active conditions, such as immune thrombocytopenic purpura or thrombotic thrombocytopenic purpura [88], making clinical interpretation difficult without confirmatory malaria diagnostics.

Biomarkers of neuronal injury, including neuron-specific enolase (NSE) and S100B [89], as well as neurofilament light chain (NFL) [90], have shown early promise in reflecting neuronal damage; however, their use remains limited to research settings and requires validation in larger cohorts. Moreover, most of these biomarkers are not routinely available in malaria-endemic LMICs due to financial and technical limitations.

### Non-invasive brain oxygenation

Near-infrared spectroscopy (NIRS) has been explored as a non-invasive tool for assessing cerebral hemodynamics in falciparum malaria patients. A study conducted by Kolyva et al. utilized NIRS to monitor spontaneous oscillations in the cerebral hemodynamics of 20 patients with CM. The findings indicated that very low-frequency oscillations were significantly higher than low- and high-frequency oscillations, with a tendency to decrease in severely ill patients, suggesting potential autonomic dysfunction in severe malaria [91].

Additionally, in another study, NIRS was used in conjunction with single-photon emission computed tomography (SPECT) to investigate cerebral perfusion and tissue oxygenation in a patient with complicated cerebral malaria. The study revealed focal right hemispheric hypoperfusion and decreased oxygen saturation, correlating with the patient’s neurological signs, and highlighting the potential of evaluating impaired cerebral microcirculation in such cases [92].

These studies suggest that NIRS can provide valuable insights into cerebral hemodynamics and oxygenation in cerebral malaria, potentially aiding in the assessment of disease severity and guiding therapeutic interventions. Despite its potential, NIRS remains largely unavailable in endemic regions due to the high cost of equipment and limited access to devices. Its integration into clinical practice is currently confined to well-resourced settings or research environments.

Table 2 summarizes the cost, feasibility, and training requirements associated with the discussed neuromonitoring tools, with a focus on their real-world applicability in LMICs.

**Table 2:** Summary of neuro-monitoring tools for cerebral malaria with considerations for use in low- and middle-income countries (LMICs)

Monitoring tool	Type	Cost	Operator training required	Feasibility in LMICs	Notes
Transcranial doppler (TCD)	Non-invasive	Moderate	High	High	Operator-dependent, may be limited by bone windows
Optic nerve sheath diameter (ONSD)	Non-invasive	Moderate	Moderate	Variable	Portable, learning curve exists
Automated pupillometry	Non-invasive	High	Low	High	Very easy to learn
EEG	Non-invasive	High	High	Limited	Requires neurophysiologist. Intermittent EEG may be the only option in LMICs
NIRS	Non-invasive	High	Low–Moderate	Limited	Equipment cost limits availability
Biomarker assays	Invasive	High	Lab infrastructure needed	Limited	Not routinely feasible in endemic settings

Cost refers to the relative expense of equipment and setup. Feasibility reflects estimated accessibility in endemic settings. Operator training indicates the level of expertise required for accurate use and interpretation. Notes highlight major advantages or limitations specific to each modality

## Treatment considerations

The initial management of patients with severe malaria and CM is similar to that of patients with sepsis of any other cause [93]. Because of frequent co-existent bacterial infections in these patients, blood cultures should be done, and antibiotics should be started early in case of shock [4]. Furthermore, as well as detecting malaria parasites and assessing the degree of parasitemia, the clinician should evaluate the potential neurological impairment, organ dysfunction, hypoglycemia, hyperlactatemia, and anemia [94]. Although not routinely required when diagnostic criteria for cerebral malaria are met and common confounders excluded, a LP may be considered in selected cases of neurological dysfunction to rule out alternative diagnoses such as bacterial meningitis. Fluid resuscitation in cerebral malaria must be approached with caution, particularly in children. The FEAST trial demonstrated that aggressive fluid boluses (saline or albumin) increased mortality in critically ill African children, likely due to fluid overload and exacerbation of cerebral edema [95]. Reflecting this, the 2024 WHO guidelines contraindicate rapid bolus infusion of crystalloids or colloids in severe malaria. Instead, fluid management should focus on correcting hypoglycemia, electrolyte disturbances, and hypotension, with individualized assessment. Adults are especially vulnerable to fluid overload, while children may present with dehydration. Clinical monitoring should include peripheral perfusion and urine output. Jugular venous pressure may be of limited value, especially outside high-resource ICU settings [34].

**Fig. 4:**
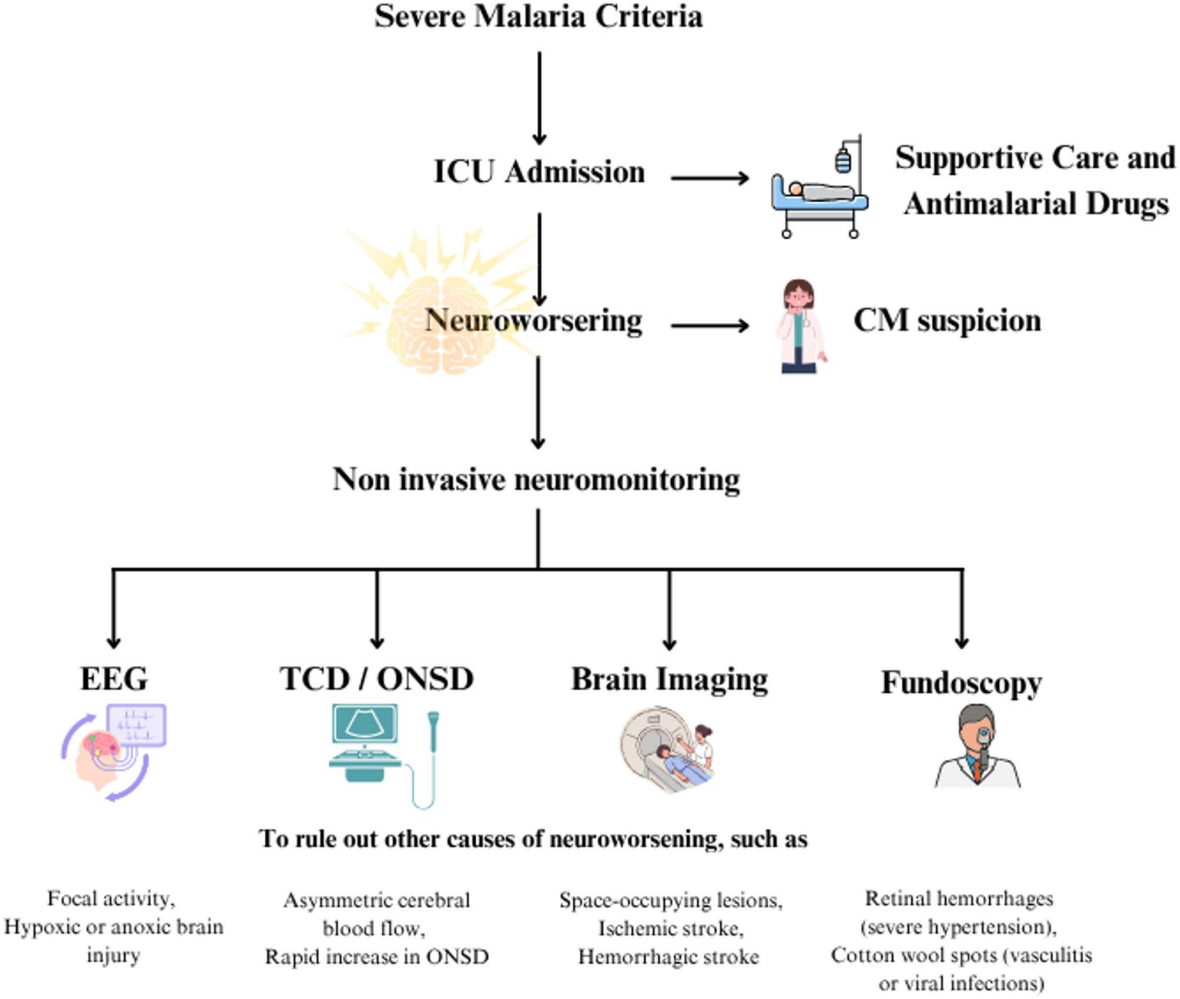
Management of severe malaria. In case of neuroworsening, monitoring of systemic variables (GHOST-CAP) is also mandatory

Treating severe CM involves using antimalarial drugs alongside supportive and targeted therapies tailored to address each affected system (Table 3). Screening for additional organ dysfunction in the ICU is also essential. Moreover, individuals with CM should be closely monitored in the critical care setting. A schematic representation of the general management of severe malaria is presented in Fig. 4.

### Neuroprotection

Apart from treating primary cerebral insults such as edema and seizures, neuroprotection in CM requires a comprehensive approach to prevent secondary brain injury [44]. Regulating glucose levels within the optimal range of 80–180 mg/dL is challenging due to the metabolic impacts of malaria [96] and medications such as artesunate [97]; however, this issue may be mitigated with intermittent glucose monitoring and modified insulin protocols in the absence of continuous monitoring. Severe anemia, a common consequence, hinders the maintenance of sufficient hemoglobin levels [98]; enhancing access to blood transfusion services or employing erythropoietin in critical cases may alleviate this issue. WHO recommends transfusion in children in high-transmission areas when hemoglobin is < 5 g/dL, and < 7 g/dL in low-transmission or non-endemic areas. These thresholds should be adapted based on clinical signs [34]. Achieving sufficient oxygenation is difficult without dependable oxygen delivery systems or monitoring; however, employing portable oxygen concentrators and emphasizing oxygen saturation monitoring with pulse oximeters might enhance outcomes [99]. Dysnatremias, including hyponatremia [100], may remain undiagnosed due to restricted access to electrolyte assessment; rectifying this may necessitate empirical correction using balanced electrolyte solutions. Addressing hyperthermia [101], intensified by malaria-related fever, poses challenges in the absence of cooling devices; nonetheless, consistent antipyretic administration and basic cooling techniques such as tepid sponging may provide viable options. These efforts underscore the necessity for adaptable, context-specific solutions to address the problems of adopting neuroprotection in CM.

### Antimalarial drugs

Regardless of the *Plasmodium species* leading to malaria, parenteral artesunate is the first-line therapy showing its superiority to quinine in several large trials [102–104] and is currently recommended for severe malaria by the WHO [34] and adopted in many national guidelines worldwide, including those of North America, which introduced it in 2019. This recommendation includes pregnant women in all trimesters and children, and treatment should never be delayed while awaiting artesunate [105].

Artesunate does not need dose adaptation concerning kidney and liver function. Nevertheless, assessing potential drug interactions due to the metabolism of artesunate by cytochrome P450 is crucial. The single dose of iv artesunate is 2.4 mg/kg at 0, 12, and 24 h. Once the patient can tolerate oral therapy and has received at least three intravenous artesunate administrations, switching to a artemisinin-based combination therapy (ACT), such as artemether/lumefantrine or dihydroartemisinin/piperaquine, is advisable. This switch should begin at least four hours after the last intravenous administration, and the oral treatment should continue for three days [4].

If the patient experienced altered mental status during the acute phase, ACTs containing mefloquine should be avoided due to the risk of neuropsychiatric adverse effects. Delayed hemolytic anemia has been reported 1–3 weeks after artesunate, particularly in patients with hyperparasitemia or in non-immune individuals. Therefore, monitoring hemoglobin after discharge is recommended [34].

Although double therapy superiority has not been demonstrated, quinine/artesunate iv association is recommended to treat severe malaria in countries where a high incidence of artesunate-resistant malaria is documented (e.g., Cambodia, Laos, Thailand, Vietnam) [106].

When intravenous quinine is used, clinicians should be aware of its potential side effects, including hypoglycemia, hypotension, and cardiac arrhythmias. Close monitoring of blood glucose levels, blood pressure, and ECG is recommended throughout the infusion period [107].

### Seizures treatment

Clinical seizures should be managed through a tiered approach. First-line treatment includes benzodiazepines such as diazepam or midazolam, which are effective in aborting seizures. The WHO recommends diazepam (0.15 mg/kg IV or 0.5 mg/kg rectal), midazolam (IM or buccal), or paraldehyde (IM) for acute seizure management [34]. Paraldehyde is rarely used in high-resource settings but remains a pragmatic option for acute seizure control in low-resource environments, particularly in children [108]. If seizures persist, a second-line antiepileptic agent such as phenytoin, levetiracetam, or valproic acid should be added. In cases of status epilepticus or refractory seizures, deep sedation with agents like propofol, midazolam, or barbiturates may be required. Refractory seizures are infrequent. A recent randomized clinical trial found that aggressive antipyretic therapy, using scheduled acetaminophen and ibuprofen, significantly reduced the incidence of multiple or prolonged seizures in children with cerebral malaria, suggesting an important role for management in seizure prevention [109].

The efficacy of prophylactic anticonvulsant drugs has been explored in three randomized trials comparing phenobarbital with either placebo or no treatment. A meta-analysis by Cochrane based on this data revealed that although anticonvulsant use was linked to a reduction in convulsions (RR, 0.30; 95% CI, 0.19–0.45), it also led to an increased risk of overall mortality (RR, 2.0; 95% CI, 1.20–3.33). This argues against the use of prophylactic phenobarbital in treating cerebral malaria [82]. No data with other antiepileptic drugs, such as leviracetam, are available.

### IH-directed therapies

In the absence of standardized protocols, IH management in CM patients may be optimized through a risk-stratified approach as already suggested in Fig. 3. Therefore high-risk patients (e.g. clinical or radiological signs of IH with or without herniation) may benefit from osmotherapy, preferably hypertonic saline, with mannitol reserved for impending herniation, while invasive monitoring or decompression should be weighed against contraindications such as thrombocytopenia; intermediate-risk patients (e.g. patients with moderate encephalopathy without clinical or radiological signs of IH) require serial non-invasive monitoring (e.g., TCD, ONSD, pupillometry) with frequent reassessment; and low-risk patients should be evaluated for alternative causes of encephalopathy (e.g., seizures, malarial retinopathy, structural lesions). Across all tiers, maintaining adequate mean arterial pressure and targeting euvolemia is essential. In low-risk patients, maintaining a MAP >65 mmHg may be sufficient [110], with higher targets (>80 mmHg) in selected intermediate- and high-risk cases. The combination of different non-invasive tools, as suggested in the B-ICONIC protocol for head trauma [111], might be a valulable alternative to integrate non-invasive neuro-monitoring tools to individualize therapeutic decisions.

Evidence on pharmacologic interventions remains limited. Dexamethasone and other corticosteroids have shown no benefit in cerebral edema reduction and are associated with an increased risk of bleeding, seizures, and delayed coma recovery [112]. Mannitol has likewise not demonstrated improved clinical outcomes in CM [113]. Although hypertonic saline may represent a potential alternative osmotic agent, evidence is scarce and largely extrapolated from other causes of raised ICP [114]. Accordingly, the WHO does not recommend corticosteroids, osmotic agents (including mannitol), or acetazolamide for CM management [34].

### Exchange transfusion

The use of exchange transfusion in malaria remains still debated. Potential advantages of this intervention include the swift elimination of circulating parasites and other compounds, such as detrimental substances in severe malaria (e.g., lactate). Additionally, exchange transfusion can address severe anemia while minimizing the risk of circulatory overload [115]. There is a lack of clinical trial data demonstrating improved outcomes associated with this intervention. Moreover, artesunate confers an almost equally rapid clearance of peripheral blood parasitemia [116]. The 2024 WHO guidelines do not recommend exchange transfusion as routine therapy, citing insufficient evidence of mortality benefit [34].

### Antimicrobial therapy

The increased susceptibility to bacterial infections in CM, particularly in pediatric patients, is attributed to immune cell exhaustion caused by the phagocytosis of parasitic byproducts, such as hemozoin. This pathophysiological mechanism highlights the importance of maintaining a high index of suspicion for secondary infections, including ventilator-associated pneumonia and sepsis, especially in critically ill patients.

Nosocomial infections represent a major complication of severe malaria [117], with ventilator-associated pneumonia being the most frequent. Antibiotic administration should be approached cautiously, particularly in hypotensive patients, children, and individuals from high-malaria-endemic areas. The selection of empiric antimicrobial therapy must be guided by local resistance patterns and adjusted based on clinical response and microbiological data.

### Adjunctive therapies

Focusing on a singular signaling pathway might not be adequate in decreasing mortality or enhancing neurological conditions in CM patients, given that CM involves multiple processes. Adjunctive therapies—defined as treatments administered in addition to standard antimalarial drugs—aim to target various pathophysiological mechanisms involved in CM. Consequently, they may help address multiple physiological processes associated with CM. Although their clinical efficacy remains under investigation, this approach is proposed to enhance clinical outcomes, extend survival, and minimize neurological damage in survivors [104]. Adjuvant therapy reduces cytoadherence and sequestration, regulates immune responses, and improves endothelial functions. In order to prioritize neuroprotection, studies have demonstrated the efficacy of adjuvant therapy in reducing mortality associated with CM in experimental cerebral malaria models. Nevertheless, clinical trial outcomes have been disheartening [118].

Modulating inflammatory response with systemic steroids, immunoglobulins, curdlan sulfate, and anti-TNF-$\:\alpha\:$ did not show a benefit and is currently not recommended for treating severe forms of malaria, including CM [119]. The other potential targets (IE sequestration and coagulation defects) are now under investigation [35], with the addition of levamisole [120] and adjunctive therapy with heparins [121].

Additional treatments have concentrated on influencing the immune response, diminishing iron levels, mitigating oxidative stress, expanding blood volume, enhancing nitric oxide production, minimizing blood coagulation, and decreasing intracranial pressure [94]. Although these interventions might prove beneficial in specific cases, the scientific literature providing robust support for their effectiveness is currently insufficient. As a result, no additional treatments are presently routinely advised in managing severe malaria.

**Table 3:** Summary of treatments for severe malaria

Category	Key points	Comments
Supportive treatment	Initial management is similar to sepsis care. Screen for organ dysfunction, start antibiotics early for co-infections, evaluate parasitemia and organ function, and use the GHOST-CAP acronym for primary brain injury.	Comprehensive screening and management are crucial in ICU settings.
Antimalarial drugs	IV artesunate as first-line therapy: 2.4 mg/kg IV at 0, 12, and 24 h, then once daily until oral therapy is possible. Followed by oral artemether/lumefantrine (20/120 mg twice daily for 3 days) or dihydroartemisinin/piperaquine (2.5 mg/kg dihydroartemisinin, 20 mg/kg piperaquine once daily for 3 days). Quinine/artesunate recommended in resistant areas.	Monitor for potential drug interactions due to artesunate metabolism.
Seizures treatment	Monitor with EEG for subclinical seizures. Use a tiered approach: (1) abort seizure with a benzodiazepine (e.g., diazepam 0.15 mg/kg IV, max 10 mg/dose, or midazolam 0.2 mg/kg IM or buccal -max 10 mg-; (2) administer an antiseizure medication (e.g., phenytoin 15–20 mg/kg IV, levetiracetam 20–60 mg/kg IV -max 4500 mg-, or valproic acid 20–40 mg/kg IV loading dose -max 3000 mg-; (3) in refractory cases, initiate deep sedation (e.g., propofol, midazolam, barbiturates**).** Prophylactic anticonvulsants such as phenobarbital are not recommended due to increased mortality risk.	Continuous EEG and clinical monitoring are key for seizure management.
Corticosteroids and mannitol	Dexamethasone and mannitol show no benefit for intracranial pressure reduction. Mannitol only in critical cases like brain herniation.	Not recommended for routine care due to risks of adverse outcomes.
Exchange transfusion	Not recommended	Not recommended
Antimicrobial therapy	High risk of bacterial co-infections (e.g., ventilator-acquired pneumonia). Early antibiotics advised, with selection based on local resistance patterns.	Target early and appropriate antibiotic use to prevent complications.
Adjunctive therapies	Focus on reducing cytoadherence, regulating immune response, and enhancing neuroprotection. Current options like steroids, heparins, and anti-TNF-α lack conclusive benefits.	Further research needed to validate therapies for routine use.

They include supportive care, antimalarial drugs, seizure management, corticosteroids and mannitol, exchange transfusion, antimicrobial therapy, and adjunctive therapies

## Prognosis

Assessing the prognosis of CM requires a multi-oriented approach that combines clinical evaluation, biomarkers, and imaging techniques. Clinically, poor outcomes are often associated with prolonged coma duration, recurrent seizures, and the presence of complications such as severe anemia, respiratory distress, circulatory failure, generalized hyporeflexia, metabolic acidosis, and hypoglycemia [122] High parasite density also correlates negatively with the prognosis [123]. In a study, the APACHE II (Acute Physiology and Chronic Health Evaluation) severity-of-disease classification system was used to stratify patients outcome with a 95.8% accurac [124]. Neurological assessments using scales such as the GCS in adults and the Blantyre Coma Scale in children are critical for determining disease severity, as lower scores correlate with worse prognosis [122, 125]. In adult patients, the 5-point coma acidosis malaria (CAM) score, using a 0–2 scoring system, involves only acidosis and mental status measured as GCS and can be accurately applied to determine the need to monitor a patient in the ICU with a CAM score >2 [126].

Biomarkers have shown promise in stratifying risk, with elevated levels of Ang-2 indicating endothelial dysfunction and poor outcomes, while HRP2 levels reflect parasite burden and blood-brain barrier disruption [127, 128]. Emerging evidence suggests that circulating red cells-derived MPs and von VWF also predict neurological injury and mortality in CM [129, 130].

Neuroimaging is essential in prognosis assessment, particularly in detecting cerebral edema, ischemia, or microhemorrhages [131]. Despite being less studied, NIRS has shown potential as a prognostic tool by assessing cerebral oxygenation and hemodynamic changes in severe malaria. Studies have indicated that NIRS can detect impaired cerebral perfusion and oxygenation, with decreased very low-frequency oscillations linked to severe cases and autonomic dysfunction [91]. These findings suggest that NIRS may help identify patients at higher risk of poor outcomes by monitoring cerebral microcirculation and oxygen delivery in real time [92]. While promising, using NIRS as a prognostic tool in CM requires further research to validate its clinical utility and integrate it into routine care.

Long-term prognosis is further influenced by the persistence of neurological sequelae, including epilepsy and cognitive deficits, which necessitate follow-up care and rehabilitation [132]. Importantly, such outcomes are not limited to severe cases alone; even less severe malaria, through persistent inflammation or immune activation, may result in lasting neurocognitive impairments [132]. Incorporating these tools into a standardized framework can improve prognostication and guide individualized treatment strategies for patients with CM.

Alongside initial neurological symptoms, some survivors of CM endure lasting neurological complications, commonly known as PMNS [40]. These consequences may present as cognitive impairments, attentional deficits, epilepsy, behavioral disorders, and motor dysfunctions, including ataxia or tremors. Research indicates that pediatric survivors of cerebral malaria are especially susceptible to neurodevelopmental abnormalities, encompassing enduring problems in memory, processing speed, and executive function [133]. The precise causes are not fully understood; however, chronic inflammation, microvascular injury, and impairment of the blood-brain barrier are believed to play a role [134].

Although various prognostic tools exist, their application in endemic areas is limited by resource constraints. Future priorities include developing accessible, point-of-care approaches that integrate clinical, biomarker, and imaging data, with neuromonitoring technologies and simplified cognitive screening, offering potential for real-time risk stratification and long-term outcome improvement, particularly in patients in LMICs.

## Conclusions

Cerebral malaria (CM) remains a life-threatening complication of severe malaria, presenting unique challenges for intensivists, particularly in low-resource settings. Despite increasing recognition of its pathophysiological complexity, no substantial evidence exists to support specific neuromonitoring strategies in adults. However, the potential implications of intracranial hypertension and cerebral perfusion disturbances demand greater attention. Management should take place in the ICU, applying general principles of intracranial pressure control and extrapolating pediatric and mechanistic data to guide neuroprotective strategies.

Looking forward, research priorities should include the development of accessible diagnostic modalities, low-cost neuromonitoring technologies, and streamlined clinical assessment protocols specifically designed for resource-limited settings. The validation of prognostic biomarkers, integration of portable neuroimaging techniques, and implementation of long-term neurocognitive follow-up should become fundamental components of CM care. Furthermore, fostering collaboration between endemic and non-endemic regions is critical to closing knowledge gaps and ensuring the adaptation of interventions to diverse epidemiological contexts. Strategic investment in these domains holds the potential to reduce CM-related mortality and mitigate its long-term neurological and socioeconomic burden.

## Data Availability

Not applicable.

## Abbreviations

ACT: Artemisinin-based combination therapy
APACHE: Acute Physiology and Chronic Health Evaluation
BBB: Blood-brain barrier
BFV: Blood flow velocity
CAM: Coma acidosis malaria
CBF: Cerebral blood flow
CDC: Centers for Disease Control and Prevention
CM: Cerebral malaria
CPP: Cerebral perfusion pressure
CSF: Cerebrospinal fluid
DIC: Disseminated intravascular coagulation
EEG: Electroencephalography
EPCR: Endothelial protein C receptor
dBFV: Diastolic blood flow velocity
GCS: Glasgow Coma Scale
HCO: Bicarbonate
ICP: Intracranial pressure
ICU: Intensive care unit
IE: Infected erythrocytes
IFN-γ: Interferon-gamma
IH: Intracranial hypertension
IL-1β: Interleukin-1 beta
LMICs: Low- and middle-income countries
LP: Lumbar puncture
MAP: Mean arterial pressure
mBFV: Mean blood flow velocity
MPs: Microparticles
MRI: Magnetic resonance imaging
NETs: Neutrophil extracellular traps
NFL: Neurofilament light chain
NIRS: Near-infrared spectroscopy
NPi: Neurological Pupil index
NSE: Neuron-specific enolase
ONSD: Optic nerve sheath diameter
PbtO2: Brain tissue oxygen pressure
PI: Pulsatility index
PMNS: Post-malarial neurological syndrome
PRES: Posterior reversible encephalopathy syndrome
RF: Respiratory Failure
RI: Resistance index
ROS: Reactive oxygen species
RR: Respiratory rate
sBFV: Systolic blood flow velocity
SBP: Systolic blood pressure
SIADH: Syndrome of inappropriate antidiuretic hormone secretion
sICAM-1: Soluble intercellular adhesion molecule-1
SPECT: Single-photon emission computed tomography
TCD: Transcranial Doppler
TMD: Tympanic membrane displacement
TNF-: Tumor necrosis factor-alpha
VWF: Von Willebrand factor
WHO: World Health Organization
